# Supplementary material for: Chromothripsis during telomere crisis is independent of NHEJ, and consistent with a replicative origin
Source: Genome Res. 2019 May;29(5):737–49. doi: 10.1101/gr.240705.118 (PMC6499312; doi:10.1101/gr.240705.118)
Supplement: Supplemental Material [file supp_gr.240705.118_Supplemental_file_1.zip › contigs/annotated_contigs/DB54/contig.2.DB54_length_930_mean_cov_11.7376344086.docx]

**DB54_length_930_mean_cov_11.7376344086**

```
GGGGGCGCACGGGGCGCCGCGGACGCGCCCCCCCCCAAGTGTCCCCGCCCCCGGGGGCGGCGGGGGGGGGGGGGGG|GGGGGAGAGAAG
                                                                             >chr19:5105
GGGGAGAGGCTTGGTGAGGGACAGGAGCCCATGTGGCCCGGGTGCTTGCCAACACCCAGGCAGCCCCATCGCCGCCTCCCTGCCCCATG
2138-51052606 + E=1e-266 p=2e-02
CCCAGAACAAAGGGACAGGGAGGGAGACAGCTGCCGATGGACCCACCCAGCAGGCTGGGGCAGGCAGAGGGAAGCTTGGCCCGCCTGGC

CCTGCACTAGCTCATCCTCTCCATGCTGGCAAGATTCTACGCCCATTCTGCAGCAGGGAAAACTGAGGCTGGGGGAGAGGAAGTGCCTG

CCCAAGGTCACTCATCCAGTAAGTGCAGAAGTTGTCCAGATTCAAAACCCAGTTCTTTGCATGACTTCAAAAAGGTAGATACATCAGAG

GAGTGAGACAGACAGACAGACAGACAAGGCGAGGGGGCAGAAACGGGGAAGAGCAAGCGAGGTGGCGAGGACCTCGCTCCCATCCCAAC

ACCCGC|CCGGT|TACTACTAATTGTGTTCGGCAGCACTGGTAGCCAGGGGAAGGCGGGGCTAGATACGATAGAGAGAGACCTTACTGA
       >chr19:50832629-50833020 + E=3e-223
ACTGAAAAAAGTGCGGGCGGGGTAGAGGGAGGAGGGGACACATCCAAGCTATTTAAAGACGCCCGACTGGCCTGGAGGGGCGGGGCCAC

CATGAGAGAGGCCGTCCTGATTGGGCTGAGAAAGGGGCGGAGAGAAACAAAACAGATCGCACGCCCTCACCTAAAACACCGCTAGGCAG

GACCTGGTGGGGAGGAGCATTGCTTGGAAGAGACAGGGCCGCAGAAAGTAGAAACGGTCCAGATGAGACTGCATGGGGCGGGAACTAGA

GATGGAGGACTTTCCCGCGCAAGGGGGACAGGCTGGACTGAAA
```
